# Disruption of nucleotide biosynthesis reprograms mitochondrial metabolism to inhibit adipogenesis

**DOI:** 10.1016/j.jlr.2024.100641

**Published:** 2024-09-06

**Authors:** Julia A. Pinette, Jacob W. Myers, Woo Yong Park, Heather G. Bryant, Alex M. Eddie, Genesis A. Wilson, Claudia Montufar, Zayedali Shaikh, Zer Vue, Elizabeth R. Nunn, Ryoichi Bessho, Matthew A. Cottam, Volker H. Haase, Antentor O. Hinton, Jessica B. Spinelli, Jean-Philippe Cartailler, Elma Zaganjor

**Affiliations:** 1 Department of Molecular Physiology and Biophysics, Vanderbilt University, Nashville, TN, USA; 2 Division of Nephrology, Department of Medicine, Vanderbilt University Medical Center, Nashville, TN, USA; 3 Creative Data Solutions, Vanderbilt Center for Stem Cell Biology, Vanderbilt University, Nashville, TN, USA; 4 Research and Medical Services, Department of Veterans Affairs, Tennessee Valley Healthcare System, Nashville, TN, USA; 5 Program in Molecular Medicine, University of Massachusetts Chan Medical School, Worcester, MA, USA; 6 Vanderbilt Digestive Disease Research Center, Vanderbilt University Medical Center, Nashville, TN, USA; 7 Vanderbilt Diabetes Research Center, Vanderbilt University, Nashville, TN, USA

**Keywords:** lipid droplets, adipocytes, nucleotides, purine, pyrimidine, adipogenesis, metabolism, fatty acid oxidation

## Abstract

A key organismal response to overnutrition involves the development of new adipocytes through the process of adipogenesis. Preadipocytes sense changes in the systemic nutrient status and metabolites can directly modulate adipogenesis. We previously identified a role of de novo nucleotide biosynthesis in adipogenesis induction, whereby inhibition of nucleotide biosynthesis suppresses the expression of the transcriptional regulators PPARγ and C/EBPα. Here, we set out to identify the global transcriptomic changes associated with the inhibition of nucleotide biosynthesis. Through RNA sequencing (RNAseq), we discovered that mitochondrial signatures were the most altered in response to inhibition of nucleotide biosynthesis. Blocking nucleotide biosynthesis induced rounded mitochondrial morphology, and altered mitochondrial function, and metabolism, reducing levels of tricarboxylic acid cycle intermediates, and increasing fatty acid oxidation (FAO). The loss of mitochondrial function induced by suppression of nucleotide biosynthesis was rescued by exogenous expression of PPARγ. Moreover, inhibition of FAO restored PPARγ expression, mitochondrial protein expression, and adipogenesis in the presence of nucleotide biosynthesis inhibition, suggesting a regulatory role of nutrient oxidation in differentiation. Collectively, our studies shed light on the link between substrate oxidation and transcription in cell fate determination.

When energy intake exceeds expenditure, organisms store surplus fuels in fat pads by increasing the size of existing adipocytes (hypertrophy) and by forming new adipocytes (adipogenesis) (1, 2). While adipocyte hypertrophy is associated with negative effects of obesity (3, 4, 5), increasing adipogenesis improves insulin sensitivity and organismal health under the challenge of excess nutrients (6, 7). Therefore, understanding the molecular mechanisms that mediate the expansion of fat depots through adipocyte hypertrophy or adipogenesis may lead to new therapies for obesity and obesity-mediated metabolic dysfunctions.

The key regulatory factors in adipogenesis include a well-defined transcriptional program composed of CCAAT/enhancer-binding proteins [C/EBPs; C/EBPα, C/EBPβ, and C/EBPδ (8)] and the peroxisome proliferator-activated receptor γ (PPARγ) (9, 10, 11, 12). In addition, studies have also revealed mitochondria to be a critical component in the regulation of adipogenesis (13). Mitochondrial expansion is induced during adipogenesis (14, 15, 16), although how this change in mitochondrial mass regulates adipocyte differentiation is poorly understood. Of note, the oxidation of pyruvate and branched-chain amino acids in the mitochondria provide a carbon source and cofactors required for lipogenesis (17, 18, 19, 20, 21). Inhibition of mitochondrial branched-chain amino acid catabolism downregulates PPARγ and consequently suppresses adipogenesis (22).

Recent studies have uncovered a novel metabolic regulation essential for supporting adipogenesis: *de novo* nucleotide biosynthesis (23). Biosynthesis of nucleotides uses nitrogen and carbon from amino acids, tetrahydrofolate derivatives, and ribose to form purines and pyrimidines. Nucleotides can be used to build cellular biomass but are also known to have a wide range of cell signaling functions. Blocking of *de novo* nucleotide biosynthesis was recently shown to suppress adipogenesis through downregulation of PPARγ (23). Therefore, we set out to identify the transcriptomic changes associated with this alteration in a critical transcription factor, with the goal of further elucidating the mechanism by which nucleotides regulate adipogenesis. We discovered that inhibition of nucleotide biosynthesis results in downregulation of transcriptional signatures associated with mitochondrial function. Morphological and functional analyses revealed that inhibiting nucleotide biosynthesis leads to altered mitochondrial cristae structure and reduced expression of mitochondrial oxidative phosphorylation (OXPHOS) proteins. Restoration of PPARγ expression in the presence of nucleotide biosynthesis inhibitors rescued mitochondrial OXPHOS protein expression. Inhibition of nucleotide biosynthesis also resulted in reprogramming of mitochondrial metabolism, including reduced tricarboxylic acid (TCA) cycle intermediates and elevated mitochondrial fatty acid oxidation (FAO). These observations prompted us to ask whether blocking FAO could rescue adipogenesis in the presence of nucleotide biosynthesis inhibitors. We found that pharmacological inhibition of FAO restored expression of PPARγ, mitochondrial proteins, and adipogenic markers, suggesting that positive feedback between intermediates of mitochondrial catabolism and the PPARγ transcriptional program regulates adipogenesis. In summary, this research sheds new light on the role of nucleotides in the regulation of mitochondrial metabolism and adipogenesis.

## Materials and Methods

### Primary SVF isolation

Primary preadipocytes were isolated from the subcutaneous white adipose tissue (WAT) of 4-to-6-day-old neonatal mice. The experimental procedures were approved by the Vanderbilt University Sub-Committee on Animal Research Care (IACUC, Institutional Animal Care and Use Committee) as required by the Public Health Service (PHS) Policy on Humane Care and Use of Laboratory Animals. WAT was digested in Hanks Buffered Saline Solution containing 1 mg/ml Collagenase Type II (Sigma, Cat #C6885), 3% bovine serum albumin (Sigma, Cat # A1470), and calcium and magnesium for 30 min at 37°C with shaking at 300 rpm. The digested tissue was washed and separated through a 100 μm cell strainer with Dulbecco’s Modified Eagle Medium (DMEM)/high glucose without sodium pyruvate supplemented with 10% fetal bovine serum (FBS), 10 μM non-essential amino acids (Thermo, Cat # 11140050), 2 mM glutamine, 20 mM HEPES, and 0.1 μM mercaptoethanol (Sigma, Cat #M3148). The filtered cell suspension was centrifuged at 600× *g* for 5 min at 4°C, and cell pellets from individual animals were resuspended in the same medium and plated separately into 6- or 12-well plates at a density of 500,000 cells per plate. The growth medium was subsequently changed every other day until the cells reached 100% confluency, at which point differentiation was induced.

### Differentiation of primary SVF cells into adipocytes

Preadipocytes isolated from WAT were transferred to an induction medium consisting of DMEM/Nutrient Mixture F-12 (DMEM/F12) with 10% FBS, 1% penicillin and streptomycin, 1.7 μM insulin, 1 μM dexamethasone (DEX), and 0.5 mM 3-isobutyl-1-methylxanthine (IBMX). The cells were kept in the induction medium for 2 days and then switched to a maintenance medium consisting of DMEM/F12 with 10% FBS, 1% penicillin and streptomycin, 17 nM insulin, 2 μM troglitazone, 1 μM rosiglitazone, and 1 nM triiodothyronine (T3). The maintenance medium was changed every other day until the endpoint of the assays.

### 3T3-L1 cell culture and differentiation

3T3-L1 cells were cultured in DMEM supplemented with 10% FBS and 1% penicillin and streptomycin. To induce differentiation, the culture medium was supplemented with a chemical cocktail of 0.5 mM IBMX, 1 μM DEX, and 1.5 μg/ml insulin. Two days after the start of induction and every 2 days thereafter, the medium was replaced with fresh culture medium supplemented with insulin.

### C3H10T1/2 cell culture and differentiation

C3H10T1/2 cells were cultured in DMEM/F12 with 1% penicillin and streptomycin and 10% FBS. Upon reaching confluency, the cells were primed to become preadipocytes by incubation for 48 h with 2.5 ng/ml BMP4. Then, differentiation was stimulated with 0.5 mM IBMX, 1 μM DEX, 10 μg/ml insulin, and 10 μM troglitazone. The cells were kept in the differentiation medium until day 2 and then switched to a maintenance medium containing 10 μg/ml insulin and 10 μM troglitazone until day 6.

### Drug treatment

Cells were treated with mizoribine (MIZ, Cayman, Cat # 23128), 5-fluorouracil (5FU, Cayman, Cat # 14416), etomoxir (ETO, Cayman, Cat # 11969) as indicated in the figure legends.

### Knockdown studies

To knockdown ADSL, shRNAs were subcloned in the pLKO.1 puro vector (Addgene Plasmid #8453) at EcoRI and AgeI sites. Primer sequences for cloning were used:

5′-CCGGCGGACCTGATTATTCTGAGAACTCGAGTTCTCAGAATAATCAGGTCCGTTTTTG-3′

5′AATTCAAAAACGGACCTGATTATTCTGAGAACTCGAGTTCTCAGAATAATCAGGTCCG-3′

Sequencing confirmed successful shRNA generation. Plasmids were transfected into platinum retroviral packaging cells (PLAT-E). Harvested lentivirus was used to infect target cells.

### Overexpression studies

PPARγ2 plasmid (Addgene #8859) or pBabe control was transfected into PLAT-E cells. Harvested retrovirus was used to infect target cells.

### BODIPY staining and imaging

Neutral lipid accumulation was measured on day 6 of differentiation using BODIPY 493/503 (Cayman, Cat # 25892). Cells were washed with phosphate-buffered saline (PBS), fixed for 1.5 h with 4% paraformaldehyde (Fisher, Cat # 50-980-488), and washed again with PBS. BODIPY and 0.1 mg/ml DAPI (Sigma, Cat #D9542) were diluted 1:1000 and incubated with the cells for 30 min at room temperature. The cells were then washed and imaged using a fluorescence microscope (Evos M5000, Life Technologies). Images were quantified using ImageJ. Basic thresholding was used to determine the BODIPY area and the number of cells per field of view to determine the BODIPY area per cell.

### TMRE

Cells were plated in 6- or 12-well glass-bottom plates for live-cell imaging experiments. Staining medium without FBS was prepared by supplementing standard cell medium with 50 nM TMRE (ThermoFisher, Cat #T669), 500 nM MitoTracker Green (Cell Signaling Technology, Cat # 9074), and 20 mM Hoechst (ThermoFisher, Cat # 62249). Cells were washed once with sterile PBS and incubated for 30 min at 37°C in a staining medium. Cells were then washed three times with sterile PBS, and the medium was replaced with a standard medium containing FBS for imaging. Quantification of the TMRE:MitoTracker Green ratio was performed using ImageJ. TMRE and MitoTracker Green signals were independently thresholded. MitoTracker Green positive regions were identified, and the TMRE positive area within these MitoTracker Green positive regions was measured. TMRE:MitoTracker Green ratios were calculated by dividing the TMRE positive area by the MitoTracker Green positive area.

### Immunofluorescence

Primary cells were grown on acid-washed coverslips in 6-well plates. After 6 days of differentiation, cells were washed with Hanks' balanced salt solution and fixed for 1.5 h with 2% paraformaldehyde (Electron Microscopy Sciences, Cat # 15710). After fixation, cells were washed with PBS and permeabilized with 0.25% Triton in PBS for 10 min. Cells were then washed with PBS and incubated with 1% bovine serum albumin (BSA) in PBS overnight at 4°C and then with primary antibody in 1% BSA in PBS overnight at 4°C. Antibodies used for immunofluorescent imaging: PPARγ (CST, Cat # 2443), perilipin-1 (CST, Cat # 9349), HA-Tag (CST, Cat # 2367). After primary incubation, the cells were washed with PBS and incubated with a secondary antibody overnight at 4°C. The next day, the cells were washed three times with PBS, and the coverslips were mounted to slides using ProLong Gold with DAPI (Invitrogen, Cat #P36931) and allowed to cure for 24 h prior to imaging. Images were acquired using a Nikon Spinning Disk confocal. Quantification of the nuclear PPARγ:DAPI ratio was performed using ImageJ. Nuclei were identified using thresholding of the DAPI signal, and the corresponding integrated densities of DAPI-positive regions were recorded. Subsequently, the integrated density of the PPARγ signal within DAPI-positive regions was recorded. Nuclear PPARγ:DAPI ratios were calculated by dividing the PPARγ integrated density by the DAPI integrated density.

### Western blotting

Cells were washed twice with PBS and lysed with RIPA lysis buffer [1% NP40, 150 mM NaCl, 25 mM Tris base, 0.5% sodium deoxycholate, 0.1% SDS, 1% phosphatase inhibitor cocktails #2 and #3 (Sigma), 1 cOmplete protease inhibitor tablet (Sigma)]. Protein content was quantified using BCA Assay (Thermo Scientific) with an equal amount of protein run on 4-20% Tris-Glycine Gels (Invitrogen). Protein was transferred to a nitrocellulose membrane (BioRad). Membranes were incubated with primary antibodies overnight at 4°C: Total OXPHOS antibody kit containing ATP5a, UQCRC2, mtCO1, SDHB, NDUFB8 (Abcam, Cat # ab110413), vinculin (Santa Cruz, Cat # sc-25336), PPARγ (CST, Cat # 2443S), C/EBPα (CST, Cat # 8178S), FABP4 (CST, Cat # 21205), perilipin-1 (CST, Cat # 9349). The following secondary antibodies were used at 1:10,000: IRDye® 800CW Donkey Anti-Mouse IgG (H + L) (Li-Cor, Cat # 925–32212), IRDye® 680RD Donkey Anti-Rabbit IgG (H + L), (Li-Cor, Cat # 926–68073). Blots were imaged with the Li-Cor Odyssey CLx infrared imaging system and are representative of at least two independent experiments.

### RNA isolation and RT-PCR

RNA was extracted with the Quick-RNA MiniPrep kit (Zymo Research). Complementary DNA was synthesized from 1 μg of RNA using the iScript complementary DNA synthesis kit (Bio-Rad). Real-time quantitative PCR was performed on a Bio-Rad CF×96 using SsoAdvanced Universal SYBR Green SuperMix (Bio-Rad). Mouse quantitative PCR primer sequences used are listed.

*Adipoq*
  Forward: TGGGATCGTAATGCACCGAG
  Reverse: GGACTGACTTGGTGGAGTGG
*Pparg*
  Forward: GTGCCAGTTTCGATCCGTAGA
  Reverse: GGCCAGCATCGTGTAGATGA
*Plin1*
  Forward: GGGACCTGTGAGTGCTTCC
  Reverse: GTATTGAAGAGCCGGGATCTTTT
*Ucp1*
  Forward: ACTGCCACACCTCCAGTCATT
  Reverse: CTTTGCCTCACTCAGGATTGG
*Pgc1a*
  Forward: CCCTGCCATTGTTAAGACC
  Reverse: TGCTGCTGTTCCTGTTTTC
*Prdm16*
  Forward: CAGCACGGTGAAGCCATTC
  Reverse: GCGTGCATCCGCTTGTG

### Seahorse assay

Basal respiration was quantified using the Agilent Seahorse XF24 Extracellular Flux analyzer. One day prior to the assay, the FluxPak was hydrated in a humidified, non-CO_2_ incubator at 37°C. Non-buffered minimal media (XF DMEM (pH 7.4) + 1 mM pyruvate + 2 mM glutamine + 1 mM glucose) and assay media (XF DMEM (pH 7.4) + 1 mM pyruvate + 2 mM glutamine + 10 mM glucose) were prepared. Two hours before the assay, the cells were washed with 1 ml of minimal media, and 500 μl of minimal media was added to each well. The cells were placed in a humidified non-CO_2_ incubator for 30 min. The long baseline protocol was selected without a mixing step to avoid perturbing the cells. The fluxpak was placed into the XF24 and the calibration step was performed. The cells were then switched to the assay media and the calibration plate was replaced with the XF24 cell culture plate containing cells. Oxygen consumption rates (OCRs) were measured every 2 min for 6 min and total of 6 cycles.

### FACS sorting

SVF cells were isolated from neonatal mice and pooled into a 10-cm dish. Media was refreshed until cells reached 100% confluency (∼4 days). Once confluent, cells were counted or prepared for western blotting and BODIPY imaging assays (total SVF). The remaining cells were used for FACS sorting. A portion of the SVF was removed to generate unstained and single-color controls for CD45-APC-Cy7 (BD Biosciences, Cat # 557659) and CD31-PE-Cy7 (Invitrogen, Cat # 25-0311-82). Prior to staining, all samples, except for the unstained, were incubated with FC block (BD Biosciences, Cat # 553141) diluted 1:100 for 5 min. The remaining pooled SVF cells were stained with CD45 and CD31 (1:1,000) for 30 min at 4°C while protected from light. Cells were washed 3 times to remove excess antibodies prior to sorting. Sorted cells (CD45-/CD31-) were then plated at the same density as the total SVF and differentiated and treated with nucleotide biosynthesis inhibitors as described.

### TEM sample preparation and analysis

TEM samples were prepared as previously described (24). Briefly, cells were fixed in 2.5% glutaraldehyde, 1% paraformaldehyde, and 120 mM sodium cacodylate for 1 h. After washing, the samples were incubated on ice for 30 min in 2% osmium solution, washed in bi-distilled H_2_O, and incubated overnight in 1% uranyl acetate solution at 4°C. The samples were then incubated in lead nitrate and aspartic acid at 60°C for 20 min and washed. Then, the samples were incubated in sequential ethanol solutions until 100% ethanol was reached. The samples were then incubated in increasing rations of Epon:ethanol until 100% Epon was reached, at which point the cells were incubated for 4 h. The embedded samples were placed on aluminum mounts in a 60°C oven for 2 days before cutting.

### Fatty acid oxidation assay

Pulsing was performed in serum-free medium containing 1 mM carnitine, 100 μM palmitate, 100 μM oleic acid, 50 μM BSA and 0.75 μCi [9,10(*n*)-^3^H] palmitic acid (GE Healthcare) for 3 h. The medium was collected and eluted in columns packed with DOWEX 1X2-400 ion-exchange resin (Sigma) to analyze the released ^3^H_2_O. ^3^H_2_O was measured in counts per minute (CPM) and normalized to total cellular protein using a BCA Protein Assay Kit (ThermoFisher Scientific).

### Triglyceride analysis

Cell pellets were washed, resuspended in sterile PBS, and stored at −80°C until processing. Lipids were extracted using the method of Folch-Lees (25). Triglycerides were methylated using BF3/methanol as described by Morrison and Smith (26), extracted, and analyzed by gas chromatography. Gas chromatographic analyses were carried out on an Agilent 7890A gas chromatograph equipped with flame ionization detectors and a capillary column (SP2380, 0.25 mm × 30 m, 0.20 μm film, Supelco). Helium was used as the carrier gas. The oven temperature was programmed from 160°C to 230°C at 4°C/min. Inclusion of lipid standards permitted quantitation of the amount of lipid in the sample.

### Metabolomics

Polar metabolites were extracted in ice-cold LC–MS grade 80:20 methanol:water. Lysates were vortexed and centrifuged at 16,000× *g* for 10 min at 4°C. Supernatants were dried down in a Vacufuge plus Benchtop Vacuum Concentrator. Dried pellets were stored at −80°C until they were subjected to metabolomic analysis performed as previously described (23) using a QExactive bench top orbitrap mass spectrometer equipped with an Ion Max source and a HESI II probe coupled to a Dionex UltiMate 3000 HPLC system.

### RNAseq

Total RNA was isolated using TRIzol reagent as per the manufacturer's instructions. Quality control analysis was performed using the Agilent Bioanalyzer and RNA Qubit assay. RNAseq libraries were prepared using 200 ng of total RNA and the NEBNext rRNA Depletion Kit (NEB, Cat #E6310X) as per the manufacturer’s instructions. This kit employs an RNaseH-based method to deplete both cytoplasmic (5S rRNA, 5.8S rRNA, 18S rRNA, and 28S) and mitochondrial (12S rRNA and 16S) ribosomal RNA from human, mouse, and rat total RNA preparations. The mRNA was enriched via poly-A-selection using oligoDT beads and then the RNA was thermally fragmented and converted to cDNA. The cDNA was adenylated for adaptor ligation and PCR amplified. The libraries were sequenced using a NovaSeq 6,000 with 150 bp paired-end reads, with a target of 50M reads per sample. RTA (version 2.4.11; Illumina) was used for base calling. Data are representative of three biological replicates.

### Transcriptomic analysis

Paired-end RNA sequencing reads (150 bp long) were trimmed and filtered for quality using Trimgalore v0.6.7. Trimmed reads were aligned and counted using Spliced Transcripts Alignment to a Reference (STAR) v2.7.9a with the –quantMode GeneCounts parameter against the GRCm38 (mm10) mouse genome (27). Sample read counts were normalized and differential expression was determined using DESeq2 v1.36.0 (28). Features counted fewer than five times across at least three samples were filtered out. Functional enrichment analysis by gene ontology (GO) was performed using clusterProfiler (v4.8.1) with ontologies obtained from the Gene Ontology Consortium (29, 30, 31, 32). OXPHOS-related genes were identified using the mouse MitoCarta 3.0 dataset (33).

### Quantification and statistical analysis

Details regarding the specific statistical tests, the definition of the center, and the number of replicates (n) for each experiment can be found in the figure legends. GraphPad Prism and MS Excel were used for all quantifications and statistical analyses.

## Results

### Transcriptomics reveals significant mitochondrial reprogramming that accompanies disruption of adipogenesis under the block of purine biosynthesis

**Fig. 1:**
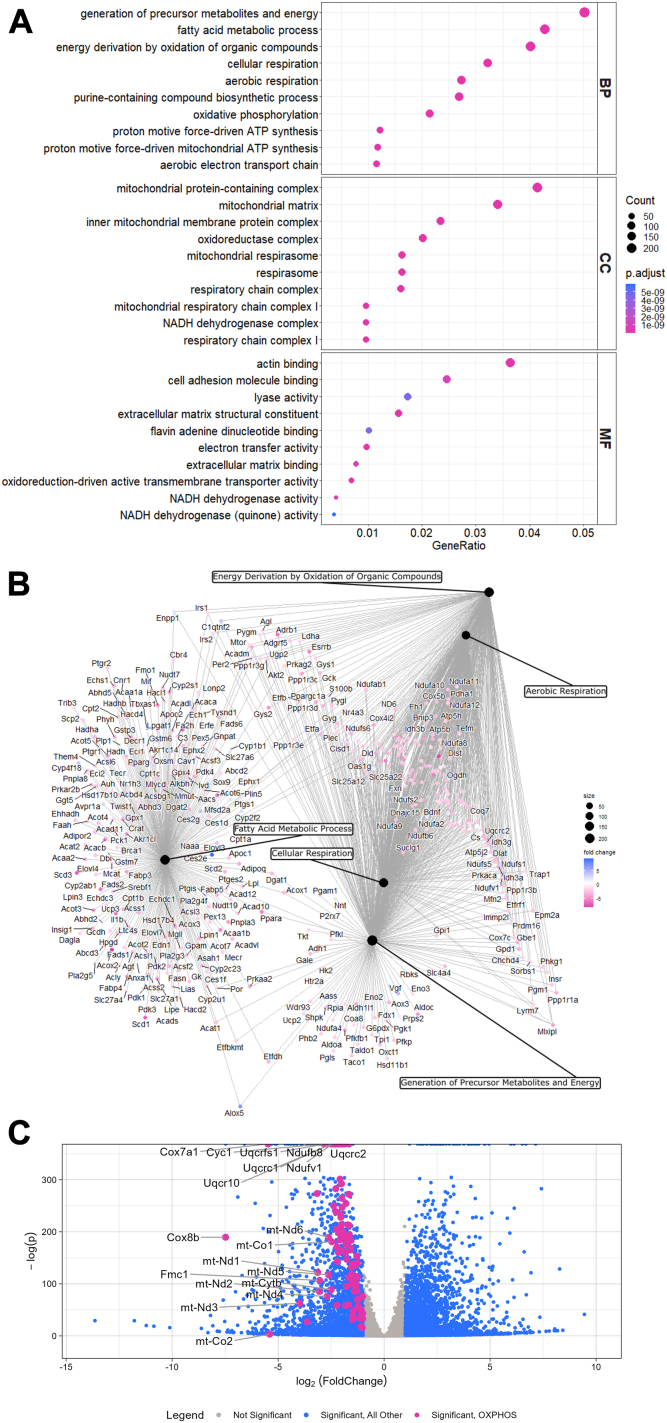
Transcriptomics reveals significant mitochondrial reprogramming that accompanies disruption of adipogenesis under the block of purine biosynthesis. A: Top 10 gene ontologies (GO) by gene ratio in the biological process (BP), cellular component (CC), and molecular function (MF) annotation categories. Size of dot represents the number of significantly (Benjamini-Hochberg adjusted *P* < 0.05), differentially expressed genes associated with each GO term. Color represents the Benjamini-Hochberg adjusted *P*-value. B: Network analysis of the top five biological processes GO terms by gene ratio. GO term node size represents the number of significantly (Benjamini-Hochberg adjusted *P* < 0.05) differentially expressed genes associated with each GO term. Gene node color represents fold change. C: Volcano plot of differentially expressed genes. Significant (Benjamini-Hochberg adjusted *P* < 0.05 and |log2(FoldChange)| > 1) differentially expressed OXPHOS-related genes (from the MitoCarta 3.0 dataset) are shown in pink. All other significantly differentially expressed genes are shown in blue, and nonsignificant genes are shown in gray. All the data in this figure were generated from the same RNA sequencing data set.

Perturbing nucleotide metabolism can alter cell identity (34, 35). Previous work has demonstrated that blocking de novo purine and pyrimidine biosynthesis inhibits adipogenesis (23). This inhibition of nucleotide biosynthesis downregulates the expression of transcriptional master regulators of adipogenesis, PPARγ and C/EBPα. However, the mechanism by which the disruption of nucleotide biosynthesis blocks these transcriptional regulators and adipogenesis remains unclear. To obtain new mechanistic insights, we examined global transcriptional changes after purine biosynthesis inhibition. Using bulk RNAseq, we compared the transcriptomes of primary stromal vascular fraction (SVF) cells that were undifferentiated or differentiated toward an adipogenic fate for 6 days in the presence or absence of a purine biosynthesis inhibitor, mizoribine (MIZ) (36), which blocks inosine monophosphate dehydrogenase 1 and 2 (IMPDH1 and 2) (Supplemental Fig. S1A). Principal component analysis (PCA) revealed tight clustering by treatment, indicating low variance among replicate samples and unique transcriptional profiles among the conditions analyzed (Supplemental Fig. S1B). To determine how the inhibition of nucleotide biosynthesis alters the transcriptomic state of the cell as compared to the differentiated adipocyte control, we examined differentially expressed genes (DEGs) and subjected them to gene ontology (GO) analysis. We identified adipogenesis as a gene set altered by MIZ, confirming our previously published studies (23) (Supplemental Fig. S1C–E). Moreover, this analysis revealed gene signatures associated with fatty acid metabolism and cellular respiration (Fig. 1A, B and Supplemental Fig. S1C). Specifically, genes coding for mitochondrial oxidative phosphorylation (OXPHOS) subunits had significantly reduced expression under MIZ treatment (Fig. 1C and Supplemental Fig. S1F). These results confirm that nucleotide biosynthesis plays a role in modulating adipocyte differentiation and suggest that the mechanism might involve mitochondrial function.

### Inhibition of nucleotide biosynthesis alters mitochondrial morphology and function

**Fig. 2:**
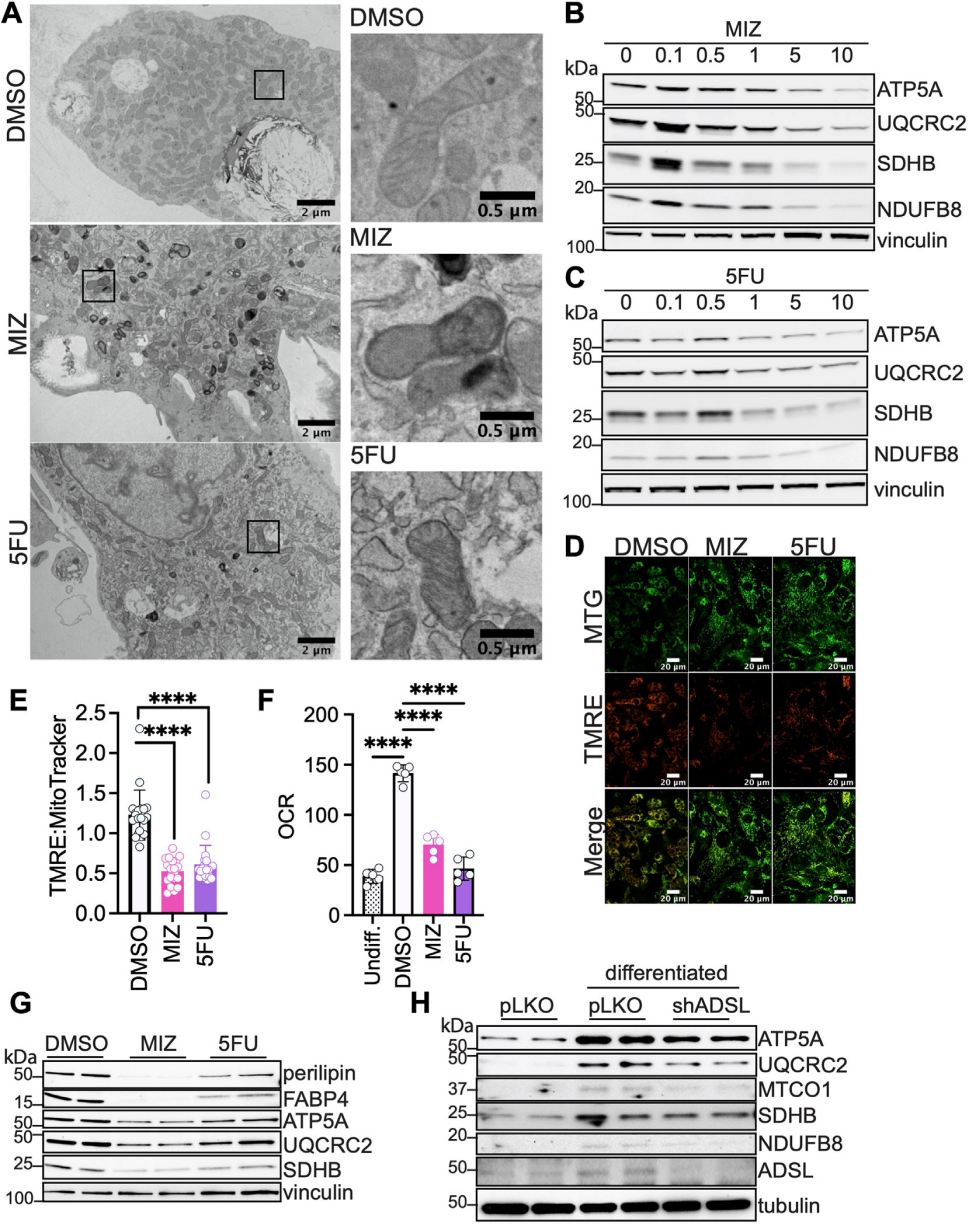
Inhibition of nucleotide biosynthesis alters mitochondrial morphology and function. A: Transmission electron microscopy was used to assess mitochondria in primary SVF cells isolated from white adipose tissue that were differentiated for 6 days in the presence of MIZ (10 μM) or 5FU (10 μM). B: Primary SVF cells were isolated and differentiated for 6 days. At the time of differentiation, cells were treated with different doses of MIZ (0, 0.1, 0.5, 1, 5, or 10 μM). On day 6 of differentiation, the cells were collected for Western blot analysis of mitochondrial OXPHOS proteins. C: SVF cells induced to adipogenesis were treated with different doses of 5FU (0, 0.1, 0.5, 1, 5, or 10 μM) and analyzed by Western blot. D: The ratio of TMRE and MitoTracker Green (MTG) staining was used to measure mitochondrial membrane potential relative to mitochondrial mass in primary SVF cells that were differentiated and treated with 10 μM MIZ or 5FU for 6 days. E: Twenty images per treatment were used to quantify the difference in signal by ImageJ. Significance was analyzed using one-way ANOVA multiple comparisons test. Error bars indicate mean ± SD, ∗∗∗∗ *P* < 0.0001. F: OCR from SVF cells undifferentiated or differentiated in the presence or absence of 10 μM MIZ or 10 μM 5FU (G) C3H10T1/2 cells were differentiated in the presence or absence of 25 μM MIZ or 10 μM 5FU. Lysates were analyzed by Western blot. (H) C3H10T1/2 cells expressing shControl (pLKO) vector or shADSL were differentiated for 6 days and analyzed by Western blot.

Because inhibition of nucleotide biosynthesis caused profound changes in mitochondrial gene expression in cells induced toward adipogenesis, we investigated the impact of these transcriptional changes on mitochondrial content and function. We first confirmed that MIZ or the pyrimidine biosynthesis inhibitor 5-fluorouracil (5FU), which blocks thymidylate synthase, consequently reducing deoxythymidine monophosphate (dTMP) levels (37), suppresses adipogenesis, as shown by decreased triglyceride levels (Supplemental Fig. S2A). We next evaluated the role of nucleotide biosynthesis in mitochondrial morphology. Primary SVF cells induced toward adipogenesis were treated with dimethyl sulfoxide (DMSO) (control), MIZ, or 5FU. Transmission electron microscopy (TEM) revealed that long-term nucleotide biosynthesis inhibition resulted in morphological changes including rounded mitochondria and altered cristae structure (Fig. 2A). We next determined whether the protein expression of key mitochondrial OXPHOS subunits was also impacted. Inhibition of nucleotide biosynthesis with MIZ or 5FU led to a dose-dependent decrease in the levels of mitochondrial OXPHOS proteins, including NDUFB8 (complex I subunit), SDHB (complex II subunit), UQCRC2 (complex III subunit), and ATP5A (complex V subunit) (Fig. 2B, C). This finding suggests that active de novo nucleotide biosynthesis may be required for mitochondrial respiration. Because the expression of OXPHOS proteins was reduced, we hypothesized that the mitochondrial membrane potential (MMP) might also be suppressed as a consequence of reduced proton gradient across the mitochondrial membrane. Using tetramethylrhodamine ethyl ester (TMRE), a dye that accumulates in regions of high MMP, we found that inhibition of nucleotide biosynthesis with MIZ or 5FU resulted in significant suppression of MMP (Fig. 2D, E). Furthermore, using the Seahorse Analyzer, we determined that mitochondrial basal respiration rate is reduced in the presence of MIZ or 5FU, as indicated by the lowered oxygen consumption rate (OCR) (Fig. 2F). Because SVF cells are a heterogeneous population, we enriched for preadipocytes by sorting out CD45 and CD31 positive cells and then treated the remaining cells with MIZ and 5FU. In the preadipocyte enriched population, MIZ and 5FU inhibited adipogenesis and OXPHOS protein expression to a similar degree as in non-sorted SVF, suggesting that the phenotypes observed are likely due to direct effects of nucleotide biosynthesis inhibitors on the adipogenic progenitors (Supplemental Fig. S2B–E). Our previous studies showed that blocking nucleotide biosynthesis even after mitotic clonal expansion prevents adipogenesis, suggesting that the effect of these inhibitors on differentiation is not dependent on their blockade of proliferation. To test whether the inhibition of nucleotide biosynthesis has distinct effects on OXPHOS protein expression before or after clonal expansion, we treated the cells with MIZ or 5FU at the initiation of differentiation (day 0) or post mitotic clonal expansion (day 3). Treating the cells with MIZ or 5FU at day 0 decreased cell number as would be predicted due to their inhibitory effect on proliferation, while treating at day 3 had no effect on cell number, suggesting that the mitotic clonal expansion had already occurred (Supplemental Fig. S2F, G). We confirmed that adipogenesis was inhibited even when the cells were treated with MIZ or 5FU after mitotic clonal expansion (Supplemental Fig. S2H, I). Moreover, we found MIZ or 5FU treatment after clonal expansion resulted in lower expression of OXPHOS proteins, suggesting that this effect is also independent of proliferation (Supplemental Fig. S2J, K). To determine if our findings in primary SVF cells are broadly applicable, we repeated the experiments using C3H10T1/2 cells, a mesenchymal stem cell line that can be differentiated into adipocytes. We found that treatment with MIZ or 5FU suppressed adipogenesis in C3H10T1/2 cells as evidenced by decreased BODIPY staining and decreased expression of perilipin and fatty acid binding protein 4 (FABP4) (Supplemental Fig. S2L, M, Fig. 2G). In addition, MIZ or 5FU treatment downregulated the expression of OXPHOS proteins ATP5A, UQCRC2, and SDHB (Fig. 2G). We recapitulated the effects of pharmacological inhibition of purine biosynthesis with a genetic approach by knocking down adenylosuccinate lyase (ADSL) in C3H10T1/2 cells and inducing the cells toward adipogenesis. Loss of ADSL modestly reduced OXPHOS protein expression (Fig. 2H) and lipid accumulation (Supplemental Fig. S2N). To evaluate whether a reduction in lipid droplets observed under the inhibition of nucleotide biosynthesis is due to increased beiging, we examined the expression of *Ucp1*, *Prdm16*, and *Ppargc1a*. MIZ and 5FU also suppressed being markers, consistent with the reduced adipogenesis (Supplemental Fig. S2O, P). Collectively, these data show that inhibition of nucleotide biosynthesis significantly affects mitochondrial morphology and function.

### Temporal inhibition of nucleotide biosynthesis reveals a more robust early suppression of transcriptional regulator PPARγ than mitochondrial function

**Fig. 3:**
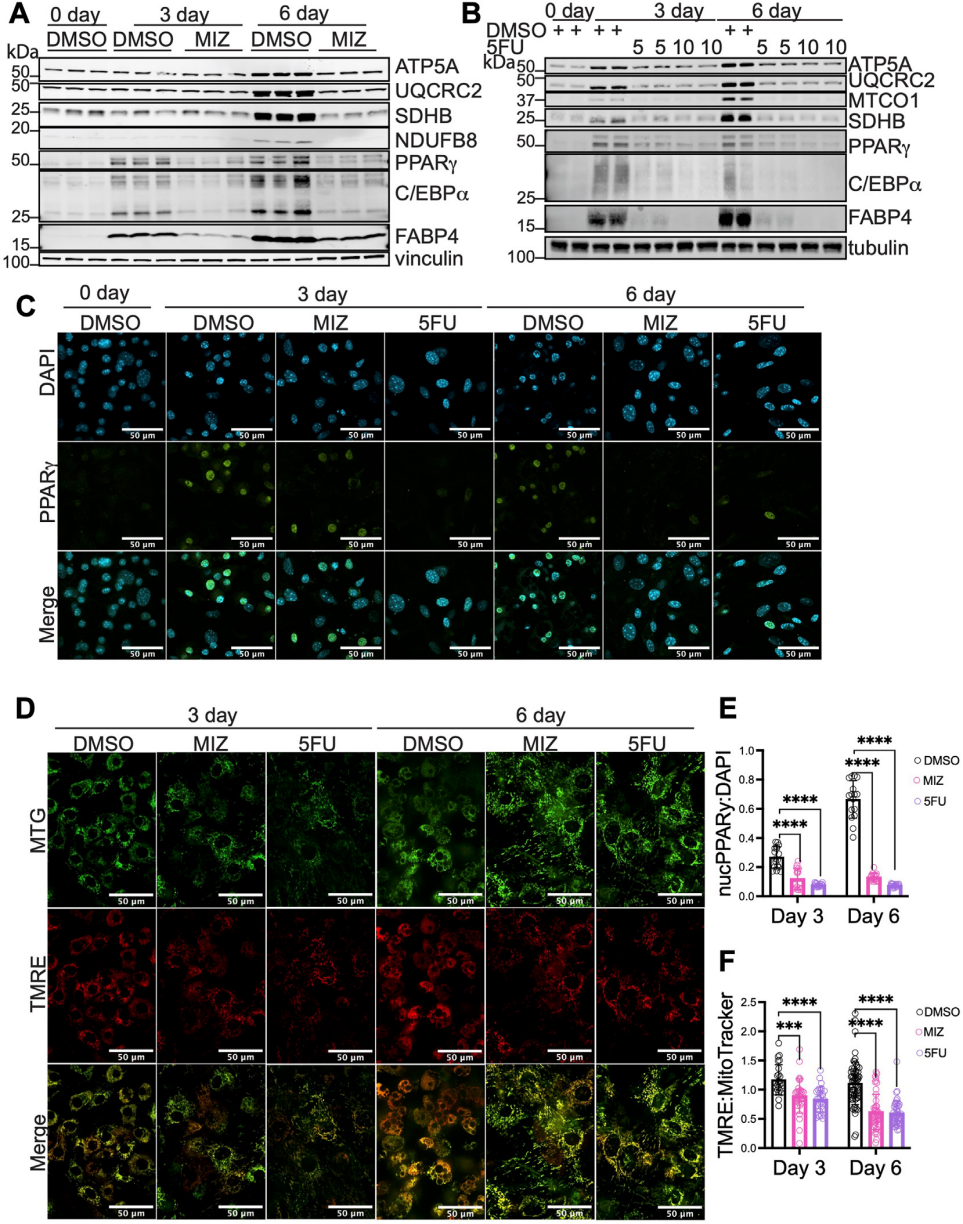
Temporal inhibition of nucleotide biosynthesis reveals a more robust early suppression of transcriptional regulator PPARγ than mitochondrial function. A: Protein levels of different OXPHOS components ATP5A, UQCRC2, SDHB, and NDUFB8 and adipogenic transcriptional regulators PPARγ (PPARg1 (53 kDa) and PPARg2 (57 kDa)) and C/EBPα (isoforms p42 and p30) in primary WAT SVF cells that were undifferentiated or differentiated for 3 or 6 days in the presence or absence of MIZ (10 μM). Data show three biological replicates. B: Protein levels of OXPHOS components ATP5A, UQCRC2, MTCO1, and SDHB, and adipogenic transcriptional regulators PPARγ and C/EBPα in primary SVF cells that were undifferentiated or differentiated for 3 or 6 days in the presence or absence of 5 μM or 10 μM 5FU. Data show biological duplicates. C: Immunofluorescence of PPARγ from SVF cells differentiated as indicated in the presence or absence of 10 μM MIZ or 10 μM 5FU. D: MTG and TMRE were used to assess changes in mitochondrial activity in primary SVF cells differentiated for 3 or 6 days in the presence or absence of 10 μM MIZ or 10 μM 5FU. Changes in (E) nuclear PPARγ or (F) TMRE were quantified using ImageJ. All experiments were repeated two or three times with two or three biological replicates. Statistical significance was determined using one-way ANOVA with multiple comparisons test. Error bars indicate mean ± SD, ∗∗∗*P* < 0.001, ∗∗∗∗*P* < 0.0001.

Blocking nucleotide biosynthesis prevents adipogenesis in part through decreased PPARγ and C/EBPα expression (23). Co-regulation has been observed between the adipogenic transcriptional program and mitochondria. Specifically, PPARγ drives mitochondrial biogenesis (16, 38), while mitochondrial oxidative amino acid metabolism supports PPARγ expression (17, 22). Therefore, we probed whether the inhibition of nucleotide biosynthesis first reduces the expression of mitochondrial proteins or transcriptional regulators. SVF cells were lysed undifferentiated or after 3 or 6 days of differentiation in the presence or absence of MIZ or 5FU. We observed that MIZ-treated cells had decreased expression of PPARγ and C/EBPα after 3 days of differentiation as compared to vehicle controls, while mitochondrial OXPHOS proteins were less affected at this time point (Fig. 3A). PPARγ, C/EBPα, and OXPHOS proteins were significantly decreased by MIZ treatment after 6 days of differentiation (Fig. 3A). Examining time points prior to 3 days of differentiation did not reveal large effects on OXPHOS protein expression or differentiation markers (Supplemental Fig. S3A). Inhibiting pyrimidine biosynthesis with 5FU more potently reduced the expression of mitochondrial proteins even after only 3 days of differentiation (Fig. 3B). Next, we examined the effects of nucleotide biosynthesis inhibition on PPARγ localization. We found that MIZ and 5FU both reduced nuclear PPARγ localization, consistent with decreased adipogenesis (Fig. 3C, E). To examine the effect of nucleotide biosynthesis inhibition on mitochondrial function early in adipogenesis, we measured MMP in the presence or absence of MIZ and 5FU. After 3 days of differentiation, MIZ and 5FU reduced MMP, indicative of decreased mitochondrial activity (Fig. 3D, F). These results suggest that although inhibition of nucleotide biosynthesis affects both transcriptional regulation and mitochondrial function, its most potent effect is on transcriptional regulation early in adipogenesis. Of note, in these studies, we cannot conclude whether inhibition of nucleotide biosynthesis prevents induction of OXPHOS proteins and transcriptional regulators or if they are induced and then depleted through a process of degradation, for example.

### PPARγ overexpression rescues mitochondrial function induced by the loss of *de novo* nucleotide biosynthesis

**Fig. 4:**
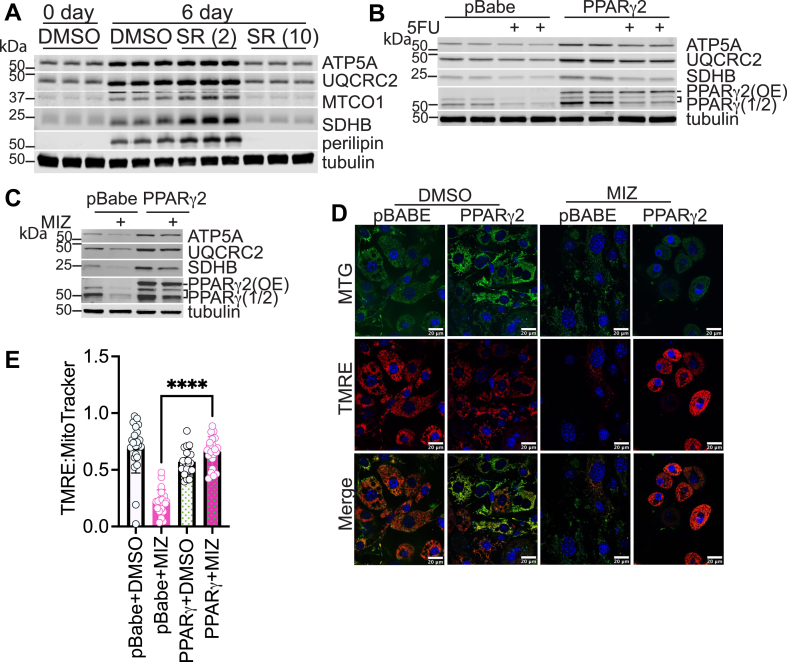
PPARγ overexpression rescues mitochondrial function induced by the loss of de novo nucleotide biosynthesis. A: OXPHOS protein levels were measured in primary SVF cells that were differentiated and treated with PPARγ inhibitor SR 16832 (2 μM or 10 μM) for 6 days (B) 3T3-L1 cells stably expressing pBABE control vector or PPARγ2 were differentiated and treated with 10 μM 5FU or DMSO (control) for 6 days. Protein expression was analyzed by Western blot as indicated. C: 3T3-L1 cells stably expressing pBABE control vector or PPARγ2 were differentiated and treated with 10 μM MIZ or DMSO (control) for 6 days. Protein expression was analyzed by Western blot as indicated. D: Live cell imaging with MTG and TMRE staining was used to assess changes in mitochondrial membrane potential relative to mitochondrial mass in the presence or absence of 25 μM MIZ. E: ImageJ was used to measure the ratio of MTG and TMRE. All experiments were repeated two or three times with two or three biological replicates. Statistical significance was determined using one-way ANOVA multiple comparisons test. Error bars indicate mean ± SD, ∗∗∗∗*P* < 0.0001.

Because PPARγ is a critical regulator of mitochondrial biogenesis in numerous systems (39) and was suppressed more robustly than mitochondrial OXPHOS proteins early in adipogenesis, we hypothesized that PPARγ expression is necessary to maintain mitochondrial protein expression. Consistent with previous studies, inhibition of PPARγ and adipogenesis with the small molecule SR 16832 reduced the expression of mitochondrial OXPHOS proteins (Fig. 4A). If PPARγ activity maintains mitochondrial protein expression, forcing PPARγ expression even in the presence of nucleotide biosynthesis inhibitors should rescue mitochondrial protein expression. Indeed, we found that expression of exogenous PPARγ2 rescued mitochondrial OXPHOS protein expression in the presence of MIZ or 5FU in 3T3-L1 preadipocyte cells that were stimulated to differentiate for 6 days (Fig. 4B, C). While it appears that PPARγ more robustly restores OXPHOS protein expression in MIZ-treated cells as compared to 5FU-treated cells, these apparent differences can likely be attributed to the level of PPARγ overexpression that was achieved in these experiments. Furthermore, overexpression of PPARγ2 restored MMP in MIZ or 5FU-treated 3T3-L1 cells, as demonstrated by increased TMRE levels (Fig. 4D and E and Supplemental Fig. S4A, B). Of note, PPARγ overexpression also restored adipogenesis in the presence of MIZ or 5FU as evaluated by restoration of lipid droplets, consistent with our published studies (Supplemental Fig. S4C) (23). These data suggest that overexpressing sufficient levels of PPARγ can mitigate the suppression of mitochondrial function and adipogenesis resulting from the inhibition of nucleotide biosynthesis.

### Inhibition of nucleotide biosynthesis blocks adipogenesis via the induction of mitochondrial fatty acid oxidation

**Fig. 5:**
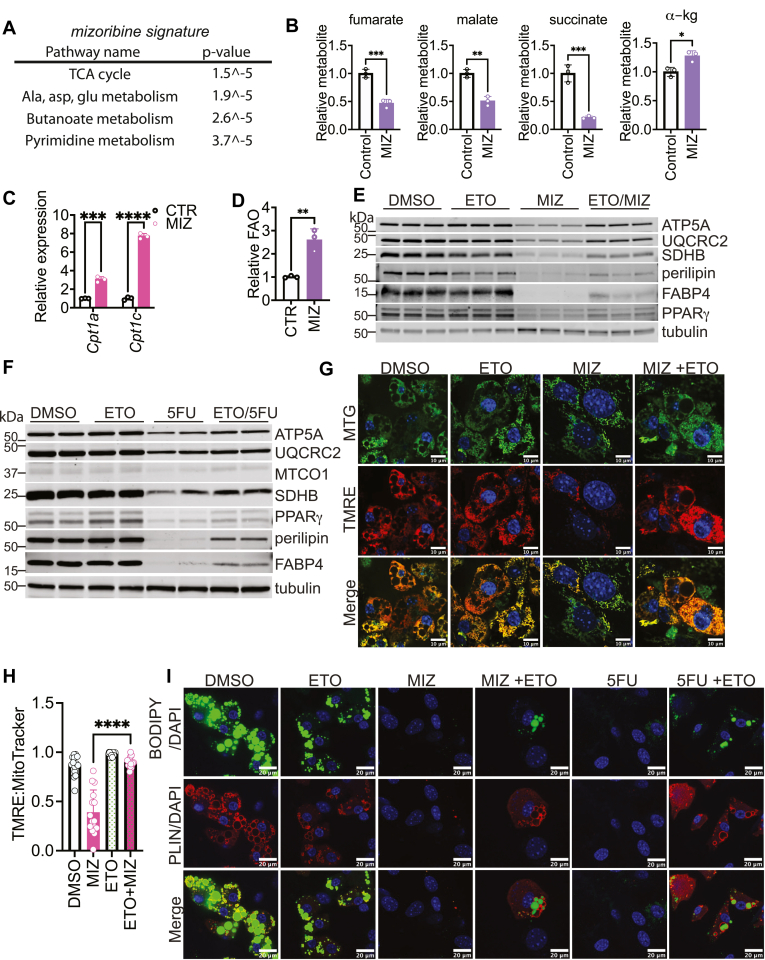
Inhibition of nucleotide biosynthesis blocks adipogenesis via the induction of mitochondrial fatty acid oxidation. A: Intracellular abundance of metabolites was profiled in 3T3-L1 cells induced to differentiate into adipocytes in the presence or absence of 25 μM MIZ. Cells were differentiated for 6 days. The pathway analysis module in MetaboAnalyst 5.0 was used to determine the most significantly altered metabolic pathways. B: Relative TCA cycle metabolite levels in 3T3-L1 cells induced to differentiate into adipocytes in the presence or absence of 25 μM MIZ. Statistical significance was determined by Student’s *t* test. Error bars indicate mean ± SD. C: Relative expression of *Cpt1a* and *Cpt1c* from bulk RNAseq described in Figure 1. Unpaired *t* test analysis. Error bars indicate mean ± SD, ∗∗∗*P* = 0.0003, ∗∗∗∗*P* < 0.0001. D: Palmitate oxidation by SVF cells stimulated to differentiate into adipocytes in the presence or absence of 25 μM MIZ. Statistical significance was determined by Student’s *t* test. Error bars indicate mean ± SD, ∗∗*P* ≤ 0.01. E: Protein expression analysis from SVF cells stimulated to differentiate into adipocytes in the presence or absence of 25 μM MIZ and/or 50 μM ETO. F: Protein expression analysis from SVF cells stimulated to differentiate into adipocytes in the presence or absence of 10 μM 5FU and/or 50 μM ETO. G: MTG and TMRE staining and live-cell fluorescent imaging from SVF cells stimulated to differentiate into adipocytes in the presence or absence of 25 μM MIZ and/or 50 μM ETO. H: ImageJ was used to analyze MTG and TMRE staining from experiments in 5G. Statistical significance was determined using one-way ANOVA with multiple comparisons test. Error bars indicate mean ± SD, ∗∗∗∗*P* < 0.0001. I: SVF cells are treated as in Figure 5G and Supplementary Figure S5C. BODIPY and PLIN were visualized by immunofluorescence.

Mitochondrial oxidative amino acid metabolism is required for the regulation of adipogenesis (17, 22, 40). To evaluate whether inhibition of nucleotide biosynthesis blocks adipogenesis by reprogramming of mitochondrial metabolism, we employed mass spectrometry to obtain steady-state metabolite profiles of 3T3-L1 fibroblasts differentiated to become adipocytes in the presence or absence of MIZ. We found that 85 out of 216 profiled metabolites were significantly altered by MIZ treatment (Supplemental Table S1). Using MetaboAnalyst 5.0, we observed that the TCA cycle is the top signature impacted by MIZ treatment (Fig. 5A). Fumarate, malate, and succinate were depleted, whereas alpha-ketoglutarate was elevated by MIZ treatment, raising the possibility that carbon flux through the TCA cycle is incomplete in the presence of nucleotide biosynthesis inhibition, which might have an impact on respiration (Fig. 5B). These data suggest that nutrient oxidation may be downregulated as a consequence of nucleotide biosynthesis inhibition, leading to the depletion of TCA cycle intermediates. We used RNAseq data from cells induced toward adipogenesis in the presence or absence of MIZ to examine gene expression in the glycolysis and branched-chain amino acid (BCAA) catabolism pathways, which are critical for adipogenesis (17, 22, 41). We found that both pathways were downregulated in the presence of MIZ (Supplemental Fig. S5A, B), whereas expression of enzymes involved in fatty acid oxidation (*Cpt1a* and *Cpt1c*) was increased (Fig. 5C). To determine if inhibition of nucleotide biosynthesis modulates fatty acid utilization, we measured the oxidation of radiolabeled palmitate. MIZ treatment increased palmitate oxidation (Fig. 5D), suggesting that nucleotide biosynthesis is involved in reprogramming mitochondrial metabolism in adipogenesis. These data led us to hypothesize that inhibition of nucleotide biosynthesis blocks adipogenesis by regulating mitochondrial fat metabolism. To determine if curbing fat oxidation would rescue adipogenesis in the presence of nucleotide biosynthesis inhibitors, we used etomoxir (ETO) to target carnitine palmitoyltransferase 1 (CPT1), a key enzyme in fat oxidation. In cells treated with MIZ or 5FU to block nucleotide biosynthesis, co-treatment with ETO partially restored the expression of OXPHOS proteins and induced a modest increase in expression of the adipogenic makers FABP4 and perilipin and increased BODIPY accumulation (Fig. 5E, F, I). Given these results, we predicted that MMP would also be rescued in cells co-treated with ETO and inhibitors of nucleotide biosynthesis. We observed that cells treated with MIZ or 5FU had reduced TMRE signal, which was restored by co-treatment with ETO, suggesting that modulating fatty acid metabolism alters MMP (Fig. 5G, H, Supplemental Fig. S5C, D). Collectively, these data suggest that inhibition of nucleotide biosynthesis reprograms mitochondrial metabolism such that increased fatty acid oxidation prevents lipid accumulation and adipogenesis.

### Nucleosides rescue the effects of de novo purine and pyrimidine biosynthesis inhibition on adipogenesis and mitochondrial protein expression

**Fig. 6:**
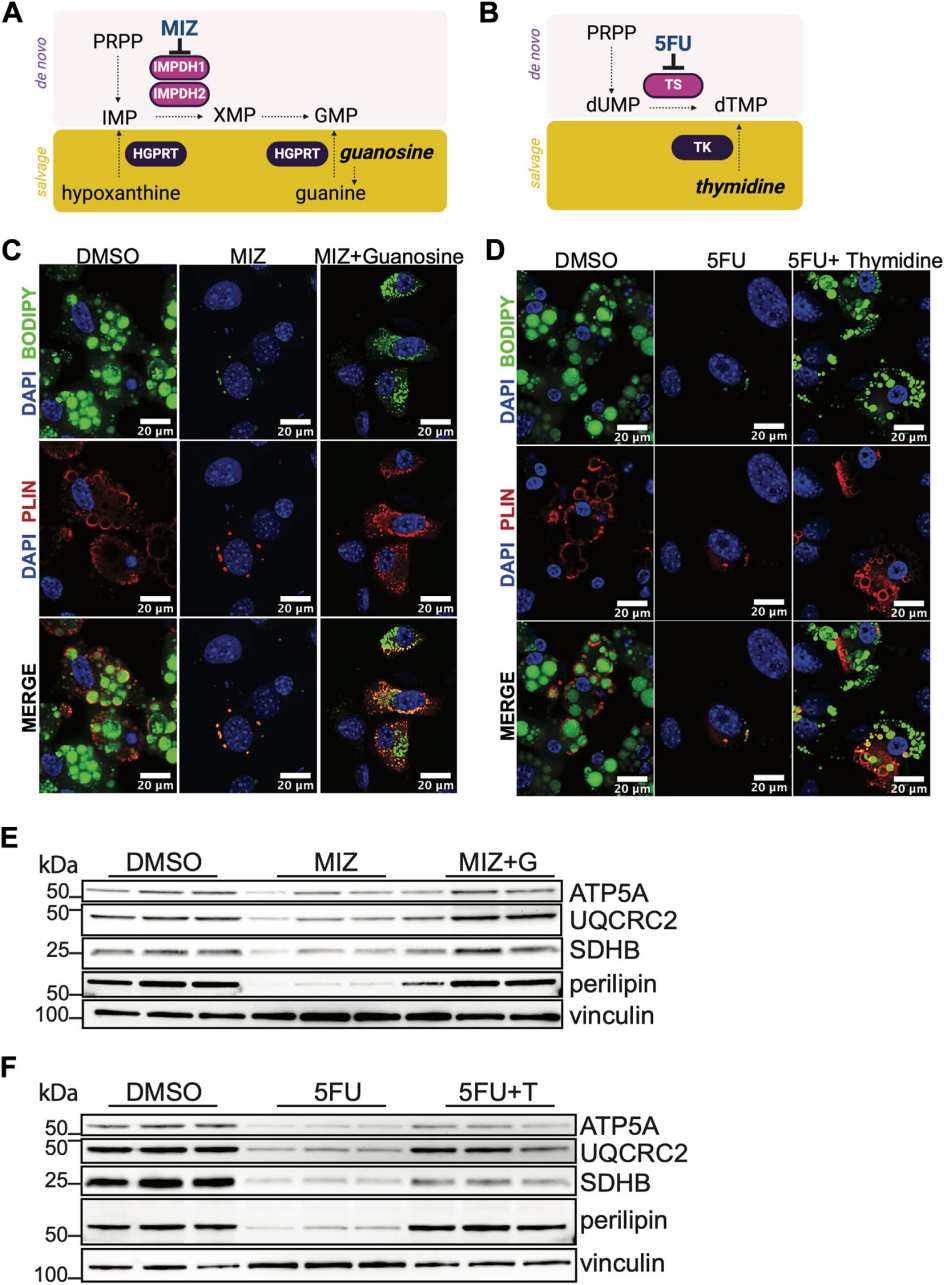
Nucleosides rescue the effects of de novo purine and pyrimidine biosynthesis inhibition on adipogenesis and mitochondrial protein expression. A: Schematic of how guanosine may rescue adipogenesis in the presence of MIZ through the purine salvage pathway. B: Schematic of how thymidine may rescue the effects of 5FU on adipogenesis. C: SVF cells stimulated to adipogenic differentiation in the presence or absence of 10 μM MIZ or 100 μM guanosine. Perilipin (PLIN), BODIPY, and DAPI were analyzed by immunofluorescence. D: SVF cells stimulated to adipogenic differentiation in the presence or absence of 5 μM 5FU or 100 μM thymidine. Perilipin (PLIN), BODIPY, and DAPI were analyzed by immunofluorescence. E: SVF cells stimulated to adipogenic differentiation in the presence or absence of 10 μM MIZ and 100 μM guanosine. Western blotting was performed as indicated. F: SVF cells stimulated to adipogenic differentiation in the presence or absence of 5 μM 5FU and 100 μM thymidine. Western blotting was performed as indicated. Data represent biological triplicates.

**Fig. 7:**
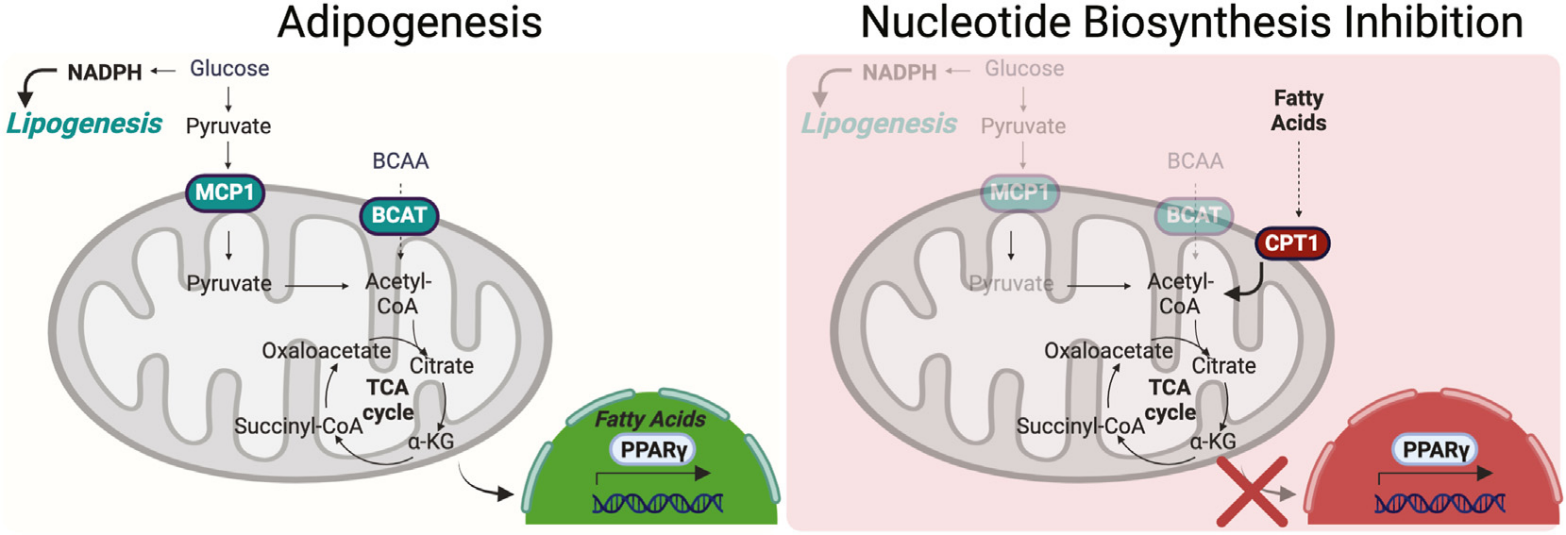
Summary model. Adipogenesis requires mitochondrial pyruvate and branched-chain amino acid (BCAA) oxidation. This metabolic program supports the expression of PPARγ. Under nucleotide biosynthesis inhibition, mitochondria increase the oxidation of fatty acids which downregulates adipogenesis. Illustration was generated using BioRender.com/l94r443.

Our previous studies indicated that the negative effects of the de novo purine biosynthesis inhibitors on adipogenesis can be rescued through the salvage pathway (23). However, we had not examined whether nucleosides can rescue adipogenesis in the presence of pyrimidine biosynthesis inhibition. To further evaluate whether exogenous nucleosides can rescue adipogenesis after purine or pyrimidine biosynthesis inhibition, primary SVF cells were exposed to MIZ or 5FU in the presence or absence of guanosine or thymidine, respectively, and adipogenesis was initiated (Fig. 6A, B). Guanosine rescued adipogenesis in the presence of MIZ, while thymidine rescued adipogenesis in the presence of 5FU, as evidenced by the restoration of lipid droplets and increased perilipin expression (Fig. 6C–F). We next examined whether adding exogenous nucleosides has any effect on mitochondrial protein expression. We observed that guanosine restored OXPHOS protein expression in the presence of MIZ (Fig. 6E). Similarly, thymidine restored OXPHOS protein expression in the presence of 5FU (Fig. 6F). Addition of excess exogenous nucleosides in the absence of nucleotide biosynthesis inhibition did not affect the expression of adipogenic or OXPHOS proteins likely indicating that the salvage pathway may not be utilized when de novo nucleotide biosynthesis is intact (Supplemental Fig. S6A, B). Altogether, these data support a model in which nucleotides are generated through the de novo nucleotide biosynthesis pathway to modulate mitochondrial metabolism and function to drive adipogenesis (Fig. 7).

## Discussion

Obesity is associated with the elevation of nucleotides in adipose tissue, although it remains unclear by which mechanism they are accumulating and the overall consequence of this accumulation (42, 43, 44). Significantly, the elevated rate of uric acid production from purine catabolism in obese adipose tissue is associated with metabolic syndrome, including glucose intolerance, dyslipidemia, and cardiovascular disease (45, 46). Therefore, valuable translational insights are likely to come from a better understanding of the role and regulation of nucleotide metabolism in adipose tissue. Previous studies have established that adipogenesis requires the induction of de novo nucleotide biosynthesis and catabolism in adipocytes, but the molecular mechanism of this regulation still requires further elucidation (47, 48).

In this study, we sought to understand the mechanism by which inhibition of nucleotide biosynthesis blocks adipogenesis. Our early experiments revealed large changes in the mitochondrial transcriptome, which prompted us to evaluate the effect of nucleotide biosynthesis on mitochondrial function and metabolism. Disruption of nucleotide biosynthesis altered mitochondrial morphology, reduced expression of proteins required for mitochondrial respiration, reduced mitochondrial activity, and induced metabolic reprogramming that resulted in increased fatty acid oxidation. Blocking fatty acid oxidation restored adipogenesis, which suggests that these changes in mitochondrial metabolism are a part of the mechanism by which inhibition of nucleotide biosynthesis impedes adipogenesis.

The temporal analysis exposed a nuanced interplay between transcriptional regulation and mitochondrial function under the inhibition of nucleotide biosynthesis. While the expression of PPARγ and C/EBPα was potently suppressed early in adipogenesis, mitochondrial OXPHOS proteins exhibited more modest downregulation. By contrast, the master transcriptional regulators and mitochondrial OXPHOS proteins were all potently downregulated by nucleotide biosynthesis inhibition later in adipogenesis. Interestingly, the inhibitory effects of blocking nucleotide biosynthesis on mitochondrial function were observed before its inhibitory effects on OXPHOS protein expression, suggesting that nucleotides regulate mitochondria through multiple mechanisms. This temporal relationship underscores the complex connection between nucleotide biosynthesis, transcriptional regulation, and mitochondrial activity during the early stages of adipocyte differentiation, which is in agreement with previously published studies (17, 22). The subsequent rescue experiments involving PPARγ overexpression provide compelling evidence that PPARγ expression can influence mitochondrial protein levels, even in the presence of stress induced by the inhibition of nucleotide biosynthesis. These findings align with existing literature that positions PPARγ as a critical facilitator of mitochondrial biogenesis during adipogenesis (16, 49, 50).

Given that restoring mitochondrial metabolism by blocking fatty acid oxidation rescued PPARγ and that restoring PPARγ expression rescued mitochondrial function in the presence of nucleotide biosynthesis inhibitors, our studies support the hypothesis that positive feedback occurs between mitochondrial metabolism and PPARγ. While regulation of mitochondrial biogenesis by PPARγ is well accepted, signaling from mitochondria to PPARγ is a relatively new observation that is poorly understood. Previous studies showed that blocking the oxidation of BCAAs in the mitochondria early in adipogenesis disrupted PPARγ activity and adipogenesis (17, 22). Indeed, these studies demonstrated that posttranslational events activate mitochondrial oxidative metabolism and respiration prior to the induction of mitochondrial biogenesis. Thus, it is possible that inhibition of nucleotide biosynthesis disrupts adipogenesis by first reducing glucose and BCAA oxidation, which provide metabolic intermediates for lipogenesis. Additionally, a block in glucose and BCAA oxidation would stimulate oxidation of fatty acids as a compensatory mechanism. Consequently, we would expect that reduced lipogenesis and increased fatty acid oxidation would result in reduced availably of fats that bind to and stabilize PPARγ (51, 52), which in turn would lead to a stall in mitochondria biogenesis. More work is required to fully test this model and elucidate which nucleotide binding enzymes in BCAA catabolism or glucose oxidation first sense changes in nucleotide availability to modulate adipogenesis.

Previous studies have also shown that mitochondria synthesize dTMP, which is required for mitochondrial DNA replication (53, 54). In the current study, we did not examine nucleotide levels in the mitochondrial compartment or the effects of 5FU on mtDNA. It is possible that this is another mechanism by which nucleotide availability impacts adipogenesis.

Our results suggest that mitochondrial fatty acid metabolism modulates PPARγ activity during adipogenesis, thus providing a new understanding of how mitochondria communicate energy state to nuclear transcriptional regulators. We propose that under normal conditions supporting adipogenesis, mitochondrial fat oxidation is restricted, leading to an accumulation of fatty acids that bind to and activate PPARγ. Conversely, when nucleotide biosynthesis is blocked, mitochondria can pivot their metabolism to consume fatty acids, thereby reducing the fatty acid-mediated activation of PPARγ. Thus, nucleotides regulate adipogenesis by modulating mitochondrial metabolism, which enables mitochondria to act as nutrient sensors that modulate fatty acid availability to influence cellular outcomes.

## Data availability

Any information required to reanalyze the data reported in this article is available from the lead contact upon request. RNAseq data files were uploaded to the Gene Expression Omnibus repository within the GSE265830 reference series.

## Supplemental Data

This article contains supplemental data

## Conflict of interest

The authors declare that they have no conflicts of interest with the contents of this article.
