# Supplementary material for: Disruption of nucleotide biosynthesis reprograms mitochondrial metabolism to inhibit adipogenesis
Source: J Lipid Res. 2024 Sep 6;65(10):100641. doi: 10.1016/j.jlr.2024.100641 (PMC11913791; doi:10.1016/j.jlr.2024.100641)
Supplement: Supplemental Figures S1–S6 [file mmc2.docx]

**Supporting Document
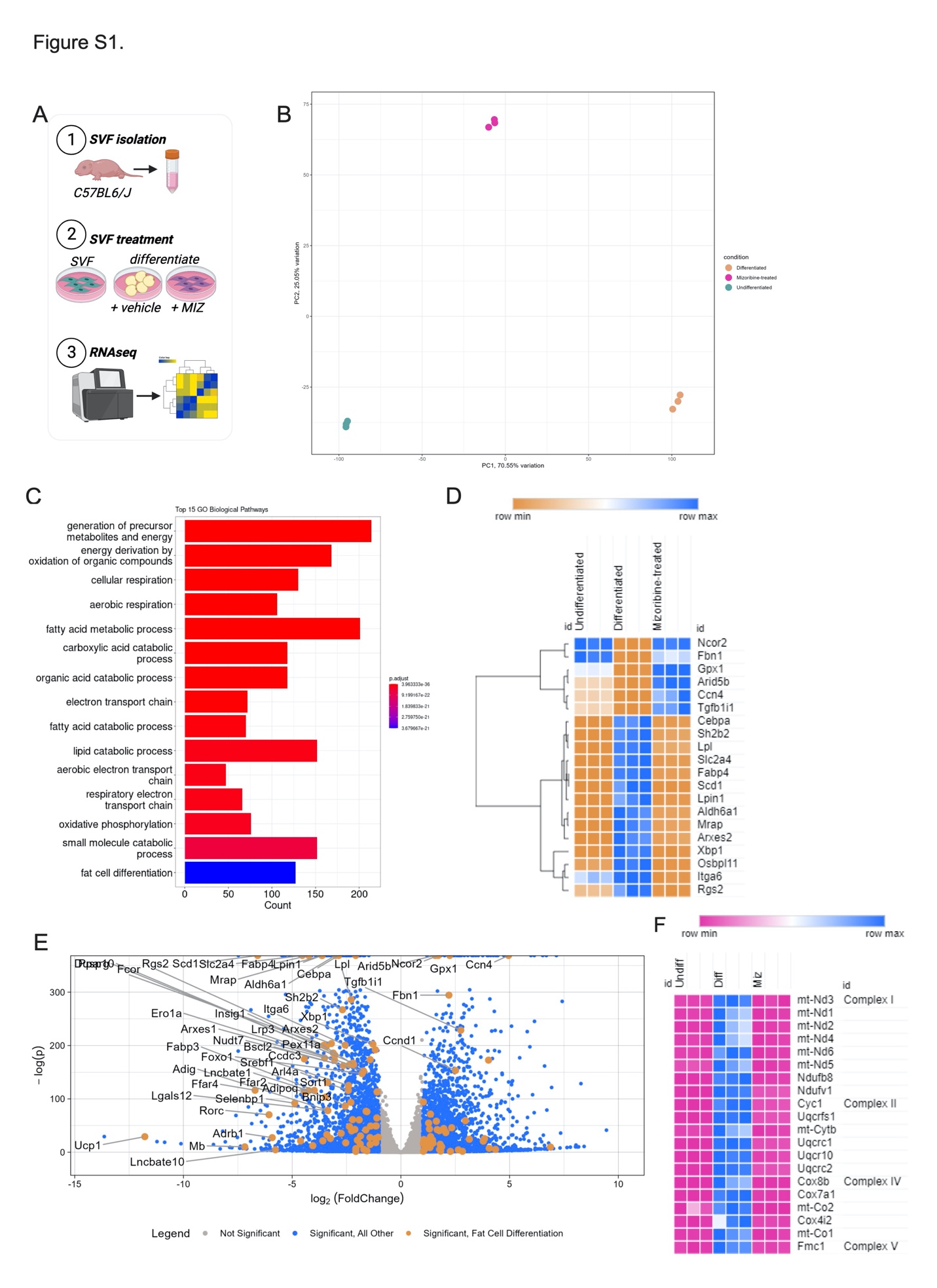
**: Pinette J.A. *et al*.

**Figure S1. Transcriptomics reveals significant mitochondrial reprogramming that accompanies disruption of adipogenesis under the block of purine biosynthesis.**

(A) Schematic of the experimental setup, primary cell isolation, differentiation, treatment, and RNA-sequencing. 3 biological replicates were analyzed for each condition. Figure S1A was created Created in BioRender. Zaganjor, E. (2023) BioRender.com/q72p095. (B) Principal component analysis (PCA) of the cellular transcriptome of primary mouse SVF cells undifferentiated or differentiated to adipocytes for 6 days and treated with either mizoribine or DMSO. (C) Top 15 GO biological pathways altered by MIZ. Data shown are from three biological replicates. (D) Expression of the top 20 genes relating to fat cell differentiation (GO:0045444) by fold change in primary mouse SVF cells differentiated to adipocytes for 6 days and treated with either mizoribine or DMSO. Hierarchical clustering by Spearman’s correlation coefficient. (E) Volcano plot of differentially expressed genes in primary mouse SVF cells differentiated to adipocytes for 6 days and treated with either mizoribine or DMSO. Significantly (Benjamini-Hochberg adjusted p < 0.05 and |log2(FoldChange)| > 1) differentially expressed genes related to fat cell differentiation (GO:0045444) are shown in orange. All other significantly differentially expressed genes are shown in blue, and nonsignificant genes are shown in gray. Data shown are from three biological replicates. The volcano plot presented is the same as in Figure 1C, from the same RNA sequencing experiment. This reuse is intended to highlight fat cell differentiation genes. (F) Gene expression of the top 20 genes related to oxidative phosphorylation (OXPHOS) by fold change. Gene annotations obtained from the MitoCarta 3.0 dataset. All the data in this figure were generated from the same RNA sequencing data set.

**
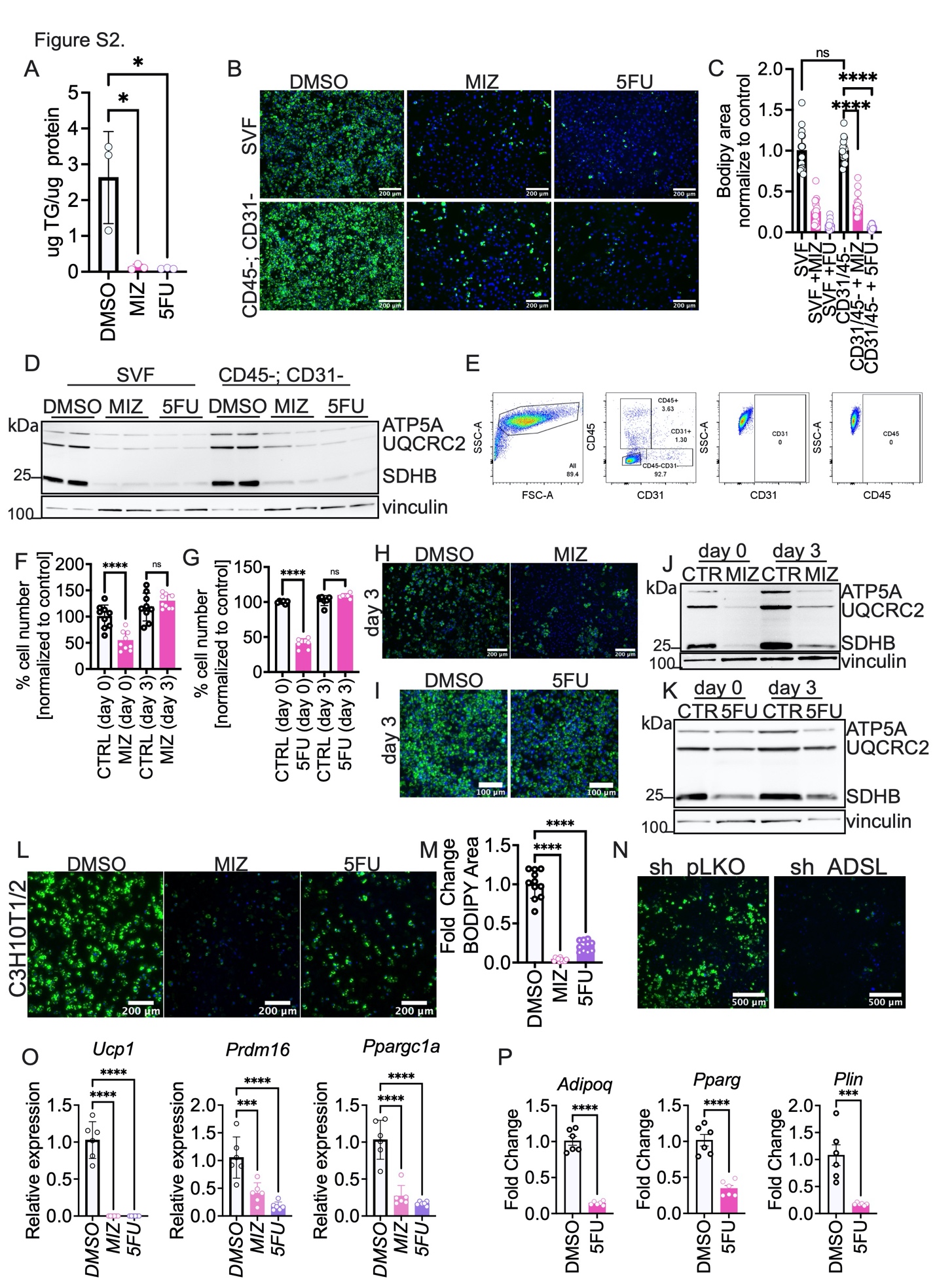
**

**Figure S2. Inhibition of nucleotide biosynthesis alters mitochondrial morphology and function.** (A) SVF cells were differentiated in the presence or absence of MIZ or 5FU (10 μM). Lipids were extracted and triglycerides were measured by gas chromatography. Statistical significance was determined using one-way ANOVA with multiple comparisons test. Error bars indicate mean +/− SD, ^∗^p < 0.05. (B) SVF cells were isolated and cultured until confluent. A portion of the total SVF was plated, and the remaining total SVF cells were FACS sorted to remove CD45+ and CD31+populations. The total SVF and CD45-/CD31- cells were differentiated in the presence or absence of MIZ (10 μM) or 5FU (10 μM). BODIPY staining was imaged. (C) Using ImageJ BODIPY area was quantified from samples in S2B. Statistical significance was determined using one-way ANOVA with multiple comparisons test. Error bars indicate mean +/− SD, ns= nonsignificant, ^∗∗∗∗^p <0.0001. (D) Protein expression of ATP5A, UQCRC2, SDHB, and vinculin were analyzed from samples treated as in S2B. Data depicts biological duplicates. (E) Gating strategy for S2B. From left to right: plot depicting total SVF without debris. Population from previous plot gated against CD45 and CD31; double negative cells were sorted. CD31 plot demonstrating that no CD31+ cells were sorted. CD45 plot demonstrating that no CD45+ cells were sorted. (F) SVF cells were differentiated and MIZ (20 μM) was added at day 0 or day 3 of differentiation. Cell number was measured by DAPI staining and quantification using ImageJ. Statistical significance was determined using one-way ANOVA with multiple comparisons test. Error bars indicate mean +/− SD, ns= nonsignificant, ^∗∗∗∗^p <0.0001. (G) SVF cells were differentiated and 5FU (5 μM) was added at day 0 or day 3 of differentiation. Cell number was measured by DAPI staining and quantification using ImageJ. Statistical significance was determined using one-way ANOVA with multiple comparisons test. Error bars indicate mean +/− SD, ns= nonsignificant, ^∗∗∗∗^p <0.0001. (H) BODIPY staining from SVF cells treated with vehicle or MIZ (20 μM) starting at day 3 post adipogenic differentiation. (I) BODIPY staining from SVF cells treated with vehicle or 5FU (5 μM) starting at day 3 post adipogenic differentiation. (J) Protein expression ATP5A, UQCRC2, SDHB, and vinculin were analyzed from samples treated as in S2H. (K) Protein expression ATP5A, UQCRC2, SDHB, and vinculin were analyzed from samples treated as in S2I. (L) C3H10T1/2 cells were differentiated for 6 days in the presence of 25 μM MIZ or 10 μM 5FU. Cells were then stained with BODIPY and imaged. (M) BODIPY area was quantified using Image J. Statistical significance was determined using one-way ANOVA with multiple comparisons test. Error bars indicate mean +/− SD, ^∗∗∗∗^p <0.0001. (N) Representative BODIPY images from control (shpLKO) and ADSL knockdown (shADSL) cells differentiated for 6 days. (O) Relative *Ucp1, Prdm16,* and *Ppargc1a* expression from SVF cells that have undergone adipogenic differentiation in the presence or absence of MIZ or 5FU. (P) Relative *Adipoq, Pparg,* and *Plin* expression from SVF cells that have undergone adipogenic differentiation in the presence or absence of 5FU.

**
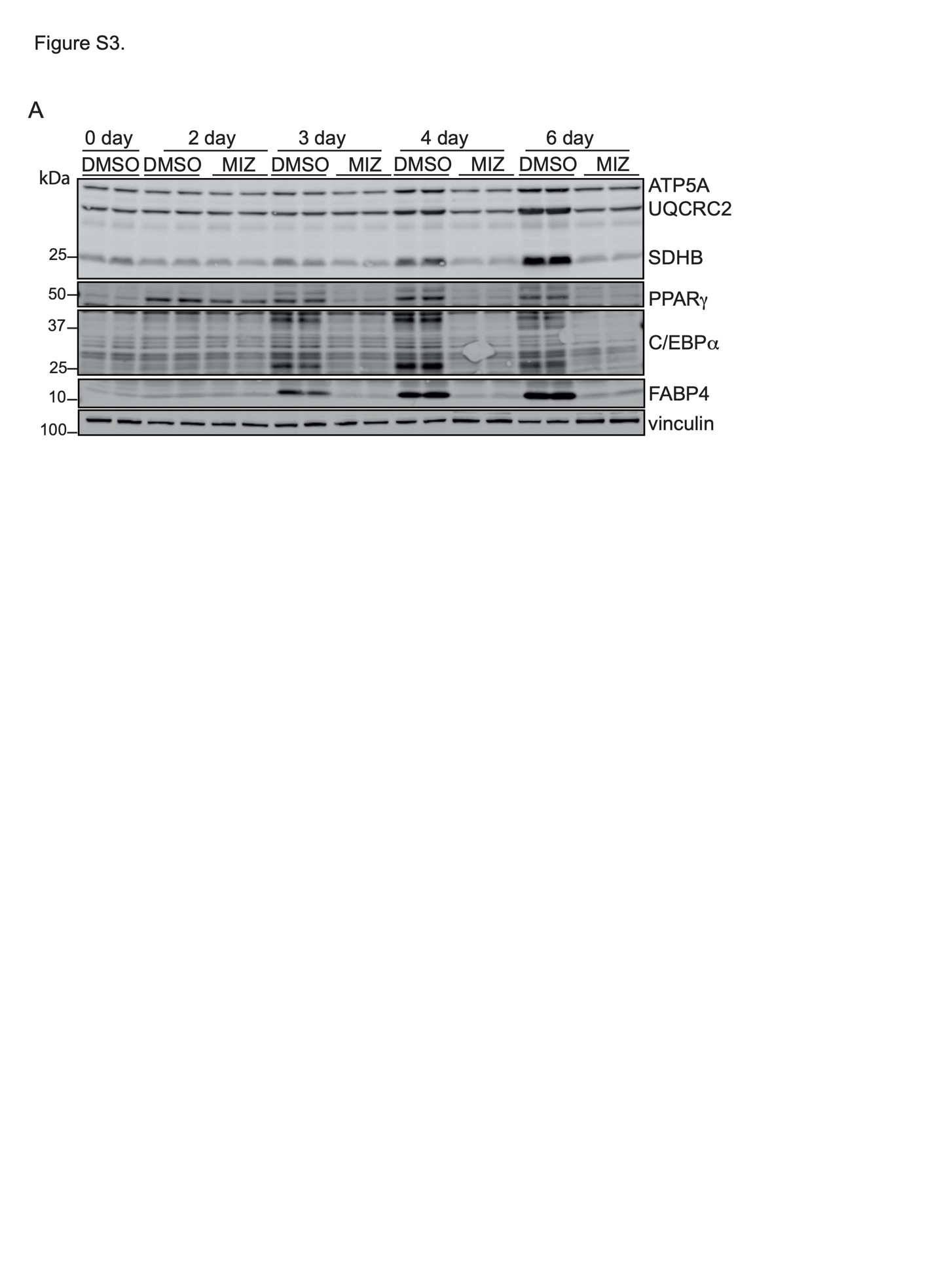
Figure S3. Temporal inhibition of nucleotide biosynthesis reveals a more robust early suppression of transcriptional regulator PPARγ than mitochondrial function.**

(A) Protein expression ATP5A, UQCRC2, SDHB, and the adipogenic transcriptional regulators PPARγ and C/EBPα in primary SVF cells that were undifferentiated or differentiated for the indicated time in the presence or absence of MIZ (10 μM).

**
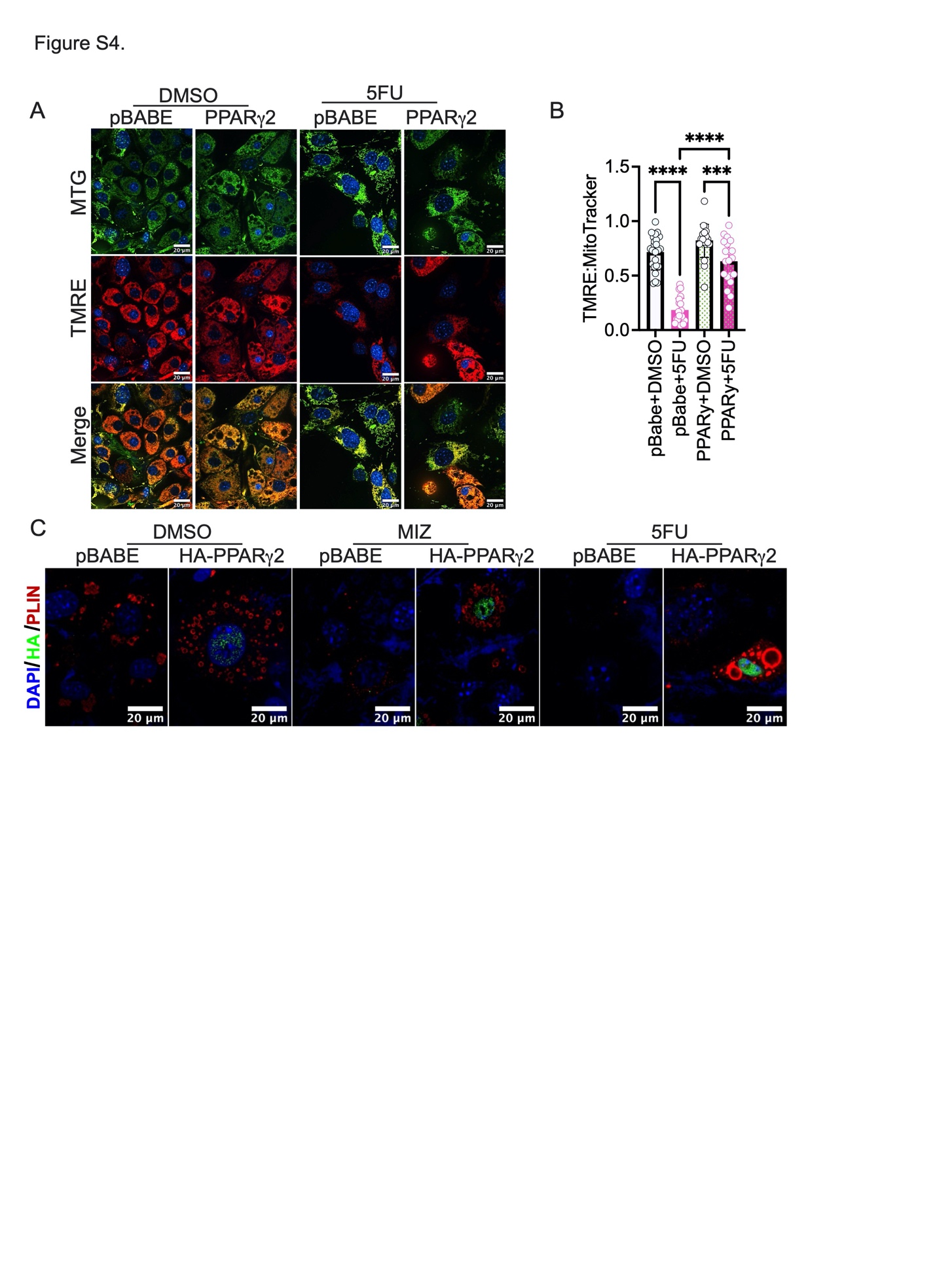
**

**Figure S4. PPARγ overexpression rescues mitochondrial function induced by the loss of *de novo* nucleotide biosynthesis.** (A) MTG and TMRE staining and live-cell fluorescent imaging from pBABE or PPARγ2 overexpressing 3T3-L1 cells stimulated to differentiate into adipocytes in the presence or absence of 10 μM 5FU. (B) ImageJ was used to quantify MTG and TMRE staining from experiment in S4A. Statistical significance was determined using one-way ANOVA with multiple comparisons test. Error bars indicate mean +/− SD, ^∗∗∗∗^p <0.0001, ^∗∗∗^p 0.0007. (C) 3T3-L1 cells stably expressing pBABE control vector or HA-PPARγ2 were differentiated and treated with DMSO (control) or 10 μM MIZ or 5 μM 5FU for 6 days. Perilipin-1 (PLIN), HA, and DAPI were analyzed by immunofluorescence.

**
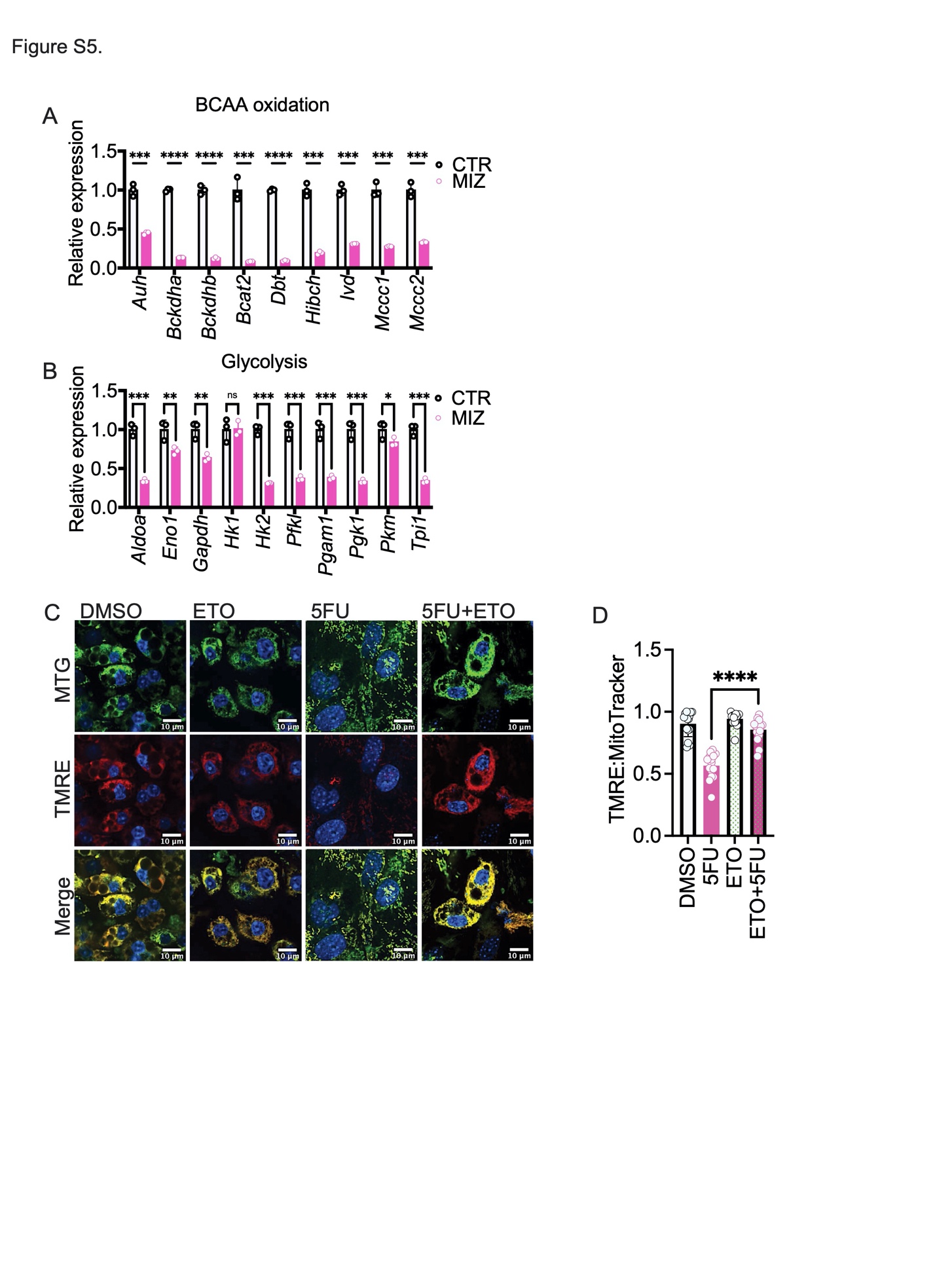
Figure S5. Inhibition of nucleotide biosynthesis blocks adipogenesis via the induction of mitochondrial fatty acid oxidation.** The RNAseq experiment described in Figure 1 was used to analyze (A) BCAA catabolic gene expression and (B) glycolytic gene expression in SVF cells differentiated in the presence or absence of 25 μM MIZ. Multiple unpaired t-tests were used to assess statistical significance. Error bars indicate mean +/− SD. (C) MTG and TMRE staining and live-cell fluorescent imaging from SVF cells stimulated to differentiate into adipocytes in the presence or absence of 10 μM 5FU and 50 μM ETO. (D) ImageJ was used to quantify MTG and TMRE staining from experiment in S5C. Statistical significance was determined using one-way ANOVA with multiple comparisons test. Error bars indicate mean +/− SD, ^∗∗∗∗^ p <0.0001.


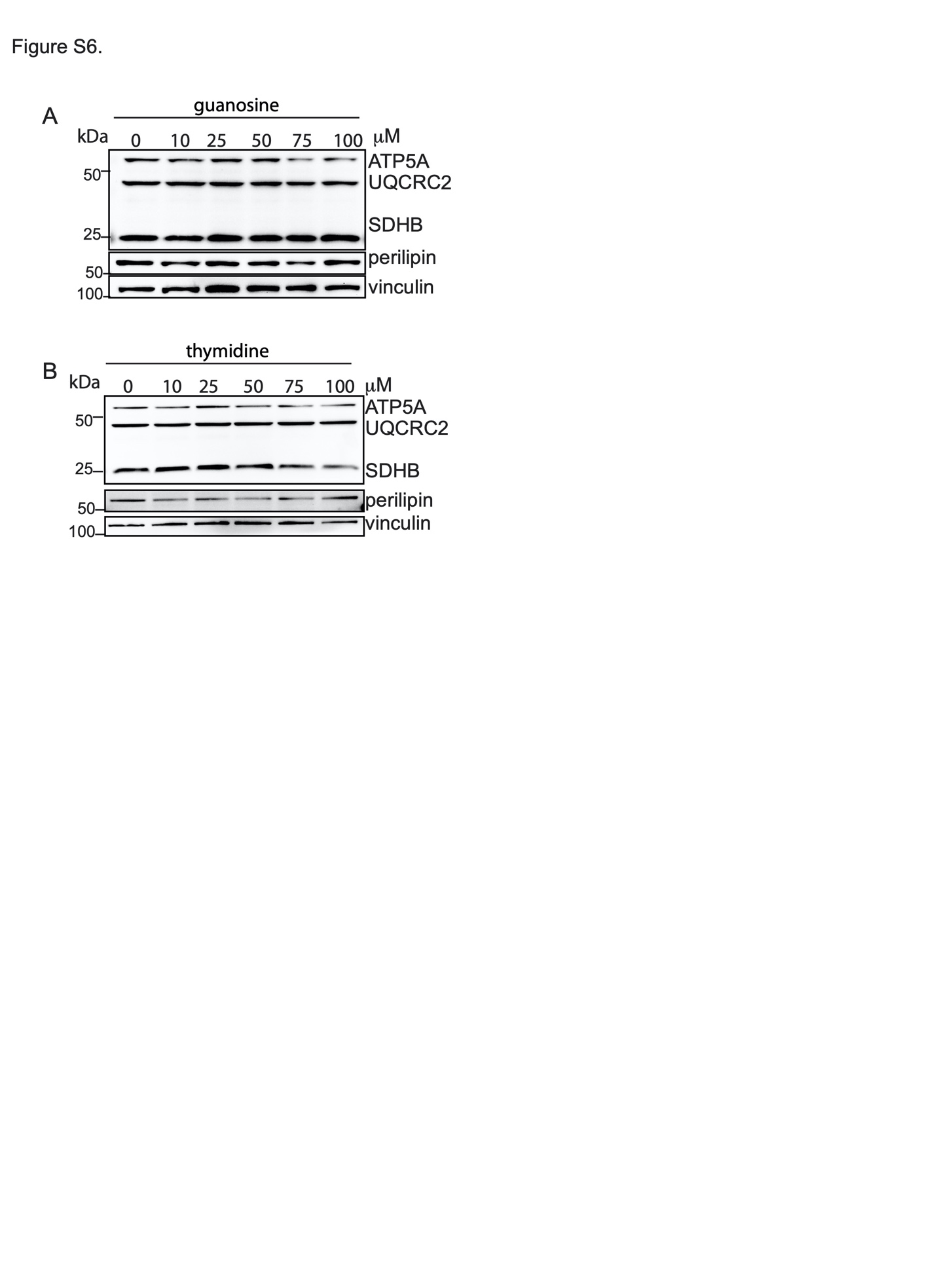


**Figure S6.** **Nucleosides rescue the effects of *de novo* purine and pyrimidine biosynthesis inhibition on adipogenesis and mitochondrial protein expression.** (A) SVF cells stimulated to adipogenic differentiation in the presence or absence of increasing μM concentrations of guanosine. Western blotting was performed as indicated. (B) SVF cells stimulated to adipogenic differentiation in the presence or absence of increasing μM concentrations of thymidine. Western blotting was performed as indicated.

**Table S1.** This supporting document corresponds to figures 5A and 5B. SVF cells were differentiated in the presence or absence of 25 μM MIZ. Metabolites were identified by mass spectrometry and their levels were normalized to cell counts.
